# Maternal fructose intake during pregnancy and lactation: Later effects on renal function

**DOI:** 10.14814/phy2.15470

**Published:** 2022-09-18

**Authors:** Leticia M. Monteiro, Celine F. Barbosa, Debora C. K. Lichtenecker, Rogério Argeri, Guiomar N. Gomes

**Affiliations:** ^1^ Laboratory of Renal Physiology, Department of Physiology, Escola Paulista de Medicina Universidade Federal de Sao Paulo Sao Paulo Brazil; ^2^ Postgraduate Program in Translational Medicine, Department of Medicine, Escola Paulista de Medicina Federal University of São Paulo São Paulo Brazil

**Keywords:** blood pressure, fructose, pregnancy, renal dysfunction

## Abstract

Excessive fructose consumption has been associated with hypertension and metabolic disorders and can alter physiological adaptations during pregnancy, with long‐term detrimental consequences. This study evaluated in post‐weaning mothers the effects of increased fructose consumption during pregnancy and lactation on blood pressure and renal function. Female Wistar rats were assigned to one of four experimental groups: non‐pregnant control (NPC); pregnant control (PC); non‐pregnant fructose (NPF), and pregnant fructose (PF). Control rats had free access to food and water, while the fructose groups had free access to food and to a 20% fructose solution, over the time period of the experiment. The systolic BP and renal function parameters were measured at the end of the experimental period, one week after weaning (28 days after delivery). The results were presented as means ± standard error. Higher values of BP were observed in both pregnant and non‐pregnant rats treated with fructose compared to control. Creatinine clearance was reduced only in the PF group; however, both the PF and NPF groups had reduced Na+ and K+ excretions. In the PF group, there was also glomerular enlargement and changes in the media/lumen (M/L) ratio of interlobular arteries. Additionally, the PF group showed increased macrophage infiltration and expression of alpha‐SM‐actin and reduced expression of nitric‐oxide‐synthase endothelial in renal tissue. These findings suggest that the association of high fructose intake with pregnancy aggravated kidney changes that persisted for up to four weeks after delivery, which may represent a risk factor for maternal health.

## Graphical abstract

Fructose overload may be harmful to maternal health due to its cardiovascular and metabolic effects. This study evaluated the effects of fructose overload during pregnancy and lactation, on maternal renal morphology and function assessed after weaning. Fructose overload altered renal function: reduced glomerular filtration rate, sodium, potassium, and urea excretion; and also caused glomerular enlargement and renal vascular alterations. These changes were observed after delivery and may have important repercussions in later life.

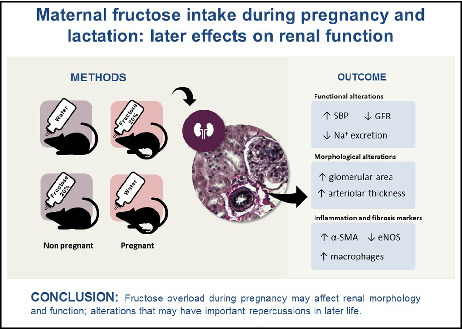

## 1 INTRODUCTION

The addition of fructose to processed foods has substantially increased since the 1970s when significant advances were made in producing this sugar. However, augmented fructose consumption has been associated with metabolic disarrangements and the development of arterial hypertension (Brito et al., 2008; De Angelis et al., 2012; Dos Santos et al., 2018; Klein & Kiat, 2015). Experimental studies have shown that fructose‐induced hypertension is associated with glucose intolerance and increased plasma levels of insulin, cholesterol, and triglycerides (Farah et al., 2006; Kamide et al., 2002). In addition, changes in the sympathetic nervous system (SNS) and the renin‐angiotensin system (RAS) also appear to contribute to fructose‐induced cardiovascular and renal changes (Cunha et al., 2007; Kamide et al., 2002; Yokota et al., 2018).

During pregnancy, anatomical and functional changes occur, including in the cardiovascular and renal systems, in an adaptive process to balance maternal and fetal needs (Odutayo & Hladunewich, 2012; Wilson et al., 2003). Usually, there is a reduction in blood pressure from the beginning of pregnancy to midterm, when it rises until 30–45 days after delivery (Rebelo et al., 2015). Renal changes occur in concert with the expansion of blood volume and the reduction of peripheral resistance, resulting in increased glomerular filtration and decreased serum levels of urea and creatinine (Wilson et al., 2003).However, gestational hypertension, which occurs in about 6% of pregnancies (Kattah & Garovic, 2013), can endanger the health of both the fetus and mother with lasting repercussions, and long‐term epidemiological studies have shown that this condition can significantly compromise renal function (Barrett et al., 2020; Vikse et al., 2008).

Nowadays, many women have postponed motherhood, which has resulted in an increase in the average age of pregnant women, with the chances of comorbidities during pregnancy being greater in older age groups (Beers & Patel, 2020). Thus, it is essential to identify factors that can interfere with gestational adaptations and bring some additional risk to its evolution.

Few studies aimed to investigate the effect of high fructose intake on maternal kidneys. Shortliffe et al. 2015 analyzed the histopathologic consequences of a high fructose diet on the kidneys of pregnant rats immediately after delivery; and observed signs of tubular damage and interstitial inflammation. Sarı et al. 2015 examined in the kidney of control and fructose‐fed dams the expression of inducible nitric oxide synthase and cyclooxygenase‐2 as markers of an early phase of inflammation; however no differences were observed between the groups. Thus, to better understand this issue, we evaluated the impact of excessive fructose consumption on blood pressure, renal morphology, and functional parameters in post‐weaning dams.

## 2 METHODS

The study was approved by the Ethical Research Committee of the Universidade Federal de Sao Paulo (protocol 7,647,020,614) and adhered to international guidelines for the care of research animals. Twelve‐week‐old female Wistar rats were obtained from the Centro de Desenvolvimento de Modelos Experimentais Para Biologia e Medicina (CEDEME) university's animal breeding center. The rats were kept in a temperature‐controlled room (22°C) with lights on from 7 am to 7 pm.

### 2.1 Experimental design

Rats were randomly assigned to the control or fructose groups. There were four experimental groups:

**NPC**: Non‐pregnant control group;

**PC**: Pregnant control group;

**NPF**: Non‐pregnant fructose group (subjected to fructose overload for a period equivalent to the pregnancy and lactation);

**PF**: Pregnant fructose group (subjected to fructose overload during pregnancy and lactation).

The control group received food and water to drink ad libitum. The fructose groups received food and a drinking solution containing 20% D‐fructose ad libitum (D‐Fructose, Labsynth, Diadema‐S.P., Brazil).

There are different experimental protocols in the literature in which the amount of fructose offered varies within a wide range (10%–60%) (Abdulla et al., 2011). The dose of 20% used in this protocol was chosen as it is relatively low, although it has been shown to exert an effect on blood pressure (Bernardes et al., 2018). One week before mating, the fructose solution was offered to the fructose group, and continued to be available during pregnancy and lactation for 24 h/day until the experimental evaluations which took place one week after weaning. Rats in the non‐pregnant fructose group had access to the solution for an equivalent period. The rats for the PC and PF groups were divided into pairs and caged overnight with a male to mate, and vaginal smears were collected the following morning. The presence of sperm was regarded as a positive result. After birth, pups were removed from any litter of more than eight so that all dams had the same size litter. The pups stayed in the dam cage for 21 days. Following weaning, the offspring were separated from the dams, placed in collective cages, and then, used in another study.

One week after weaning, the blood pressure of the dams was measured, and kidney function and morphology were evaluated.

### 2.2 Measurement of systolic blood pressure (BP)

BP was determined in conscious rats with a tail‐cuff method (IITC Life Science, Inc.). Rats were habituated to the procedure for two weeks by placing them in the heated measurement chamber (34°C) for up to 10 min. For measurement, the cuff (with a sensor connected to a recording system) was positioned on the rat's tail. The cuff was inflated to 220 mmHg and slowly deflated, and the systolic pressure was recorded. Blood pressure was calculated from four inflations/deflations in sequence in each session. Four to five sessions were performed for each rat.

### 2.3 Evaluation of renal function

The rats were placed in metabolic cages (Criffa) for 24 h. Urine and blood samples were collected to measure creatinine, urea, sodium, and potassium concentrations.

Plasma and urine creatinine levels were measured using the Jaffe method (creatinine K vet, Labtest), and the glomerular filtration rate (GFR) was determined based on creatinine clearance. Plasma and urine urea levels were measured using a urea detection kit (uréia‐CE, Labtest). The concentrations of sodium and potassium were measured using flame photometry (Analyzer 910). The quantification of urinary protein concentration was performed using the Bio‐Rad Protein Assay (Bio‐Rad Laboratories Inc).

### 2.4 Renal morphology

After functional assessments, the rats were anesthetized with ketamine (65 mg/kg) and xylazine (6 mg/kg) (with an additional dose of anesthetic administered as a bolus for cardiac arrest) and the kidneys were surgically removed and fixed in Bouin's solution (ethanol saturated with picric acid 75%, formaldehyde 20%, and acetic acid 5%), before being embedded in paraffin. Five‐micron histological sections were cut and stained with hematoxylin and eosin. The glomerular area was evaluated with a light microscope (Nikon H550L) and camera. Images were analyzed on a computer with image analysis software (Nikon, NISElements 3.2). Encircled areas were determined by computerized morphometry. Twenty fields were analyzed on each slide (magnification 200×). Kidney slides were also stained by Verhoeff and Van Gieson method for interlobular arteries analysis; thirty arteries in each slide were evaluated (magnification 400×). For immunohistochemical analysis, sections were incubated overnight at 4°C with the antibodies against: CD68 for macrophage identification (monoclonal anti ED1, dilution 1:500, Serotec, Sigma‐Aldrich); anti‐proliferating cell nuclear antigen (PCNA), dilution 1:1000, (MyBioSource); anti‐alpha‐SM‐actin, dilution 1:1000 (Dako, Glostrup‐Denmark); anti‐eNOS, dilution 1:250, (Gene‐tex) and anti‐8OHDG, dilution 1:150 (Gene‐tex). The reaction product was determined with a universal immuno‐peroxidase polymer (Histofine ‐ Nichirei Biosciences), or with Alexa‐fluor 488, dilution 1:500 (Invitrogen – Thermo Fisher Scientific). For the quantitative analysis, the percentage of the marked area was assessed in 20 consecutive fields of each sample (200× magnification). Images were acquired with a microscope (Eclipse 80i, Nikon) equipped with a digital camera (DSRi1, Nikon), and analyzed with NIS‐Elements (Nikon) software.

### 2.5 Statistical analysis

Results were presented as mean ± standard error, and analyzed by two‐way ANOVA. Additionally, Tukey's post hoc test was used for multiple comparisons between the groups (Prism 6.0, GraphPad). Values of *p* ≤ 0.05 were considered significant.

## 3 RESULTS

**FIGURE 1:**
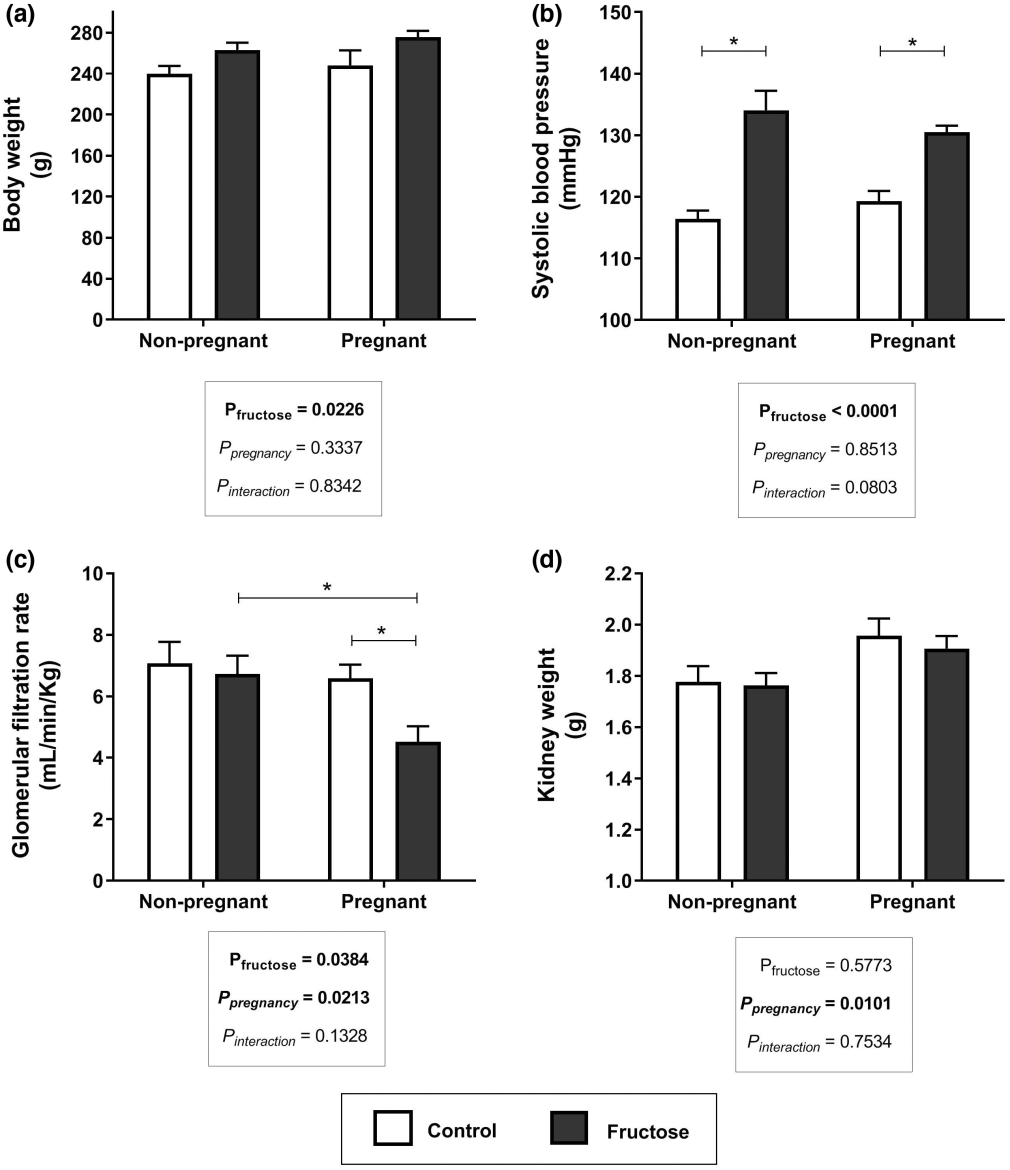
Effect of fructose overload on body weight (a), systolic blood pressure (b), glomerular filtration rate (c), and kidney weight (d) values are mean ± standard error. Tukey's post‐hoc test, *p < 0.05. The assessments were performed one week after weaning on 7–10 animals per group.

Figure 1 presents the results obtained for body weight, blood pressure, glomerular filtration rate, and kidney weight from the experimental groups. Fructose intake had a positive effect on the body weight of the pregnant and non‐pregnant rats (Figure 1a). The treatment also altered blood pressure (BP) values in both groups treated with fructose; BP in NPF was about 15% above values from NPC; in PF group it was about 10% above PC (Figure 1b). A significant reduction in the glomerular filtration rate (about 30%) was observed in the pregnant rats that received fructose compared to PC (Figure 1c). Pregnancy had a positive effect on kidney weight (Figure 1d). In Table 1 are shown the results from blood and urine analysis, and also the liquid, fluid, and caloric intake. Caloric intake was similar in all groups, even though there was a reduction in food intake in the groups that received fructose. Higher urine volume, but lower sodium, potassium, and urea excretion were observed in fructose‐treated groups. However, sodium and potassium blood concentrations remained in the normal range. The changes observed in ions excretion may have a physiological impact. There was no significant effect of fructose on creatinine blood concentration; nevertheless, urea blood concentration was significantly reduced in the groups treated with fructose.

**TABLE 1:** Summary of urinary and blood parameters observed in pregnant or non‐pregnant rats submitted to fructose overload

Parameter	Non‐pregnant		Pregnant		Two‐way‐ANOVA		
	Control (*N* = 7)	Fructose (*N* = 7)	Control (*N* = 10)	Fructose (*N* = 10)	Fructose effect	Pregnancy effect	Interaction effect
Urinary							
Urinary volume (ml/24 h)	11.4 ± 0.8	25.2 ± 2.1*	14.9 ± 0.7	26.2 ± 3.8*	***p* < 0.0001**	*p* = 0.3722	*p* = 0.6184
Proteinuria (mg/24 h)	1.4 ± 0.24	1.9 ± 0.38	1.6 ± 0.32	1.3 ± 0.29	*p* = 0.7312	*p* = 0.5504	*p* = 0.2099
Na^+^ excretion (mEq/24 h)	1.5 ± 0.14	0.7 ± 0.09*	1.4 ± 0.12	1.0 ± 0.10*	***p* < 0.0001**	*p* = 0.4951	*p* = 0.1841
K^+^ excretion (mEq/24 h)	4.2 ± 0.15	2.1 ± 0.27*	4.0 ± 0.21	2.4 ± 0.36*	***p* < 0.0001**	*p* = 0.7895	*p* = 0.3315
Urea excretion (mEq/24 h)	80.2 ± 2.7	36.8 ± 4.9*	84.6 ± 2.6	47.4 ± 7.8*	***p* < 0.0001**	*p* = 0.1286	*p* = 0.5190
Blood							
Creatinine (mg/dl)	0.37 ± 0.03	0.37 ± 0.02	0.42 ± 0.04	0.55 ± 0.06	*p* = 0.1781	***p* = 0.0210**	*p* = 0.2231
Urea (mg/dl)	41.9 ± 1.8	21.7 ± 2.5*	39.1 ± 2.6	33.6 ± 1.6^**#**^	***p* < 0.0001**	*p* = 0.0539	***p* = 0.0029**
[Na^+^]_p_ (mEq/L)	142.1 ± 1.3	140.1 ± 0.9	143.9 ± 1.2	145.6 ± 1.8	*p* = 0.9186	***p* = 0.0175**	*p* = 0.2133
[K^+^]_p_ (mEq/L)	3.9 ± 0.13	4.0 ± 0.17	4.3 ± 0.36	4.0 ± 0.18	*p* = 0.6536	*p* = 0.4563	*p* = 0.3961
Daily intake							
Liquid intake (ml/24 h)	27.8 ± 1.3	41.5 ± 3.5*	26.9 ± 3.0	35.3 ± 4.3	***p* = 0.0017**	*p* = 0.2670	*p* = 0.4088
Food intake (g/24 h)	16.1 ± 0.6	8.0 ± 0.8*	16.3 ± 1.0	11.8 ± 1.2* ^**#**^	***p* < 0.0001**	***p* = 0.0432**	*p* = 0.0635
Caloric intake (kcal/24 h)	56.4 ± 2.3	61.2 ± 4.5	57.0 ± 3.5	69.6 ± 6.5	*p* = 0.0536	*p* = 0.3000	*p* = 0.3697

*Note:* Differences statistically significant when *p* < 0.05; vs. control* or non‐pregnant^**#**^ using Tukey post test after two‐way‐ANOVA. Values are means ± standard error. *N* = number of measurements. The assessment were performed one week after weaning.

**FIGURE 2:**
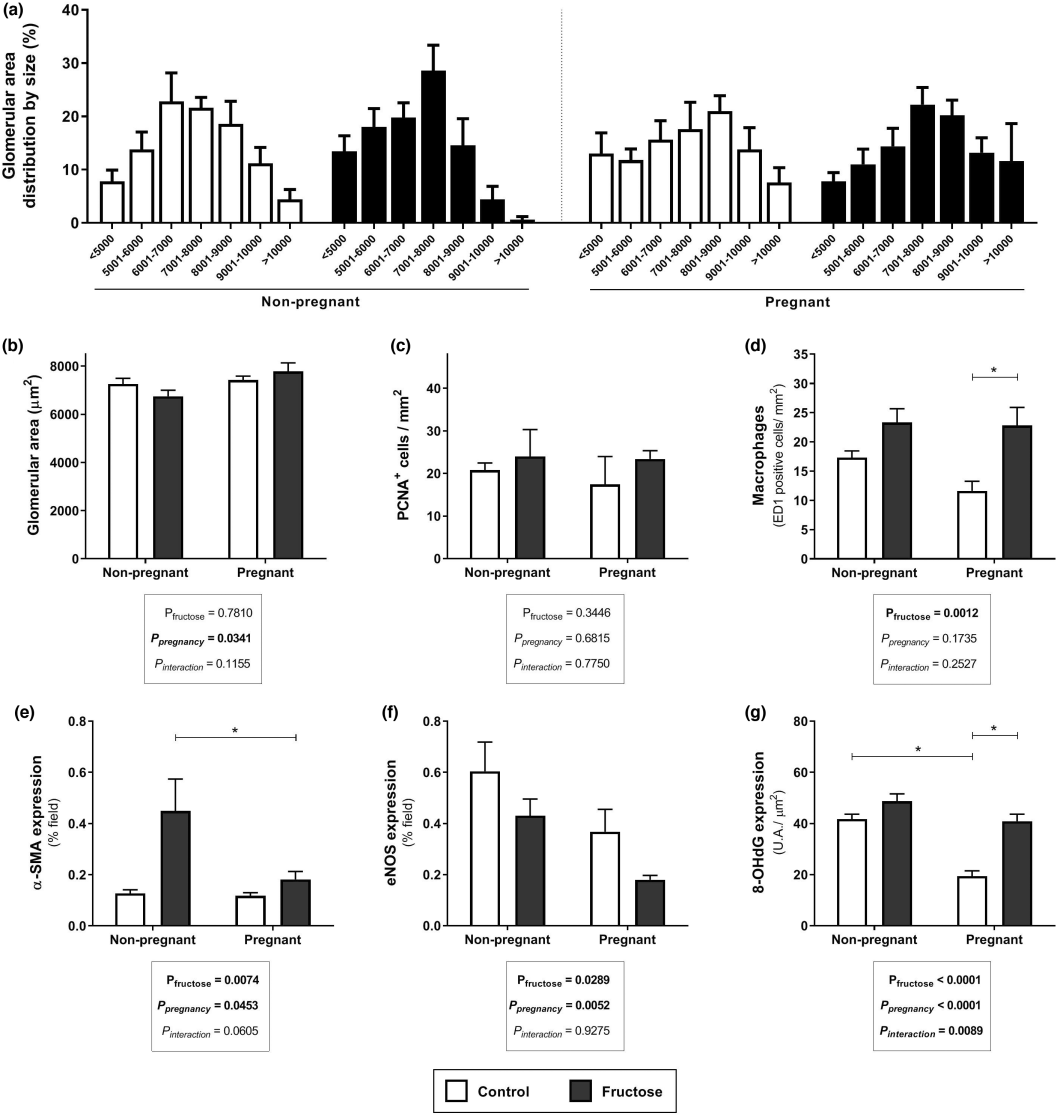
Overview of kidney morphological features and inflammatory and fibrosis markers of female rats receiving or not fructose overload. Glomerular area distributed by size (%) (a), glomerular area (b), number of PCNA positive cells (c), number of macrophages (d), expression of α‐SMA (e), eNOS (f), and 8‐OHdG (g) values are mean ± standard error. Tukey's post‐hoc test, *p < 0.05. The assessments were performed one week after weaning on 5–6 animals per group.

**FIGURE 3:**
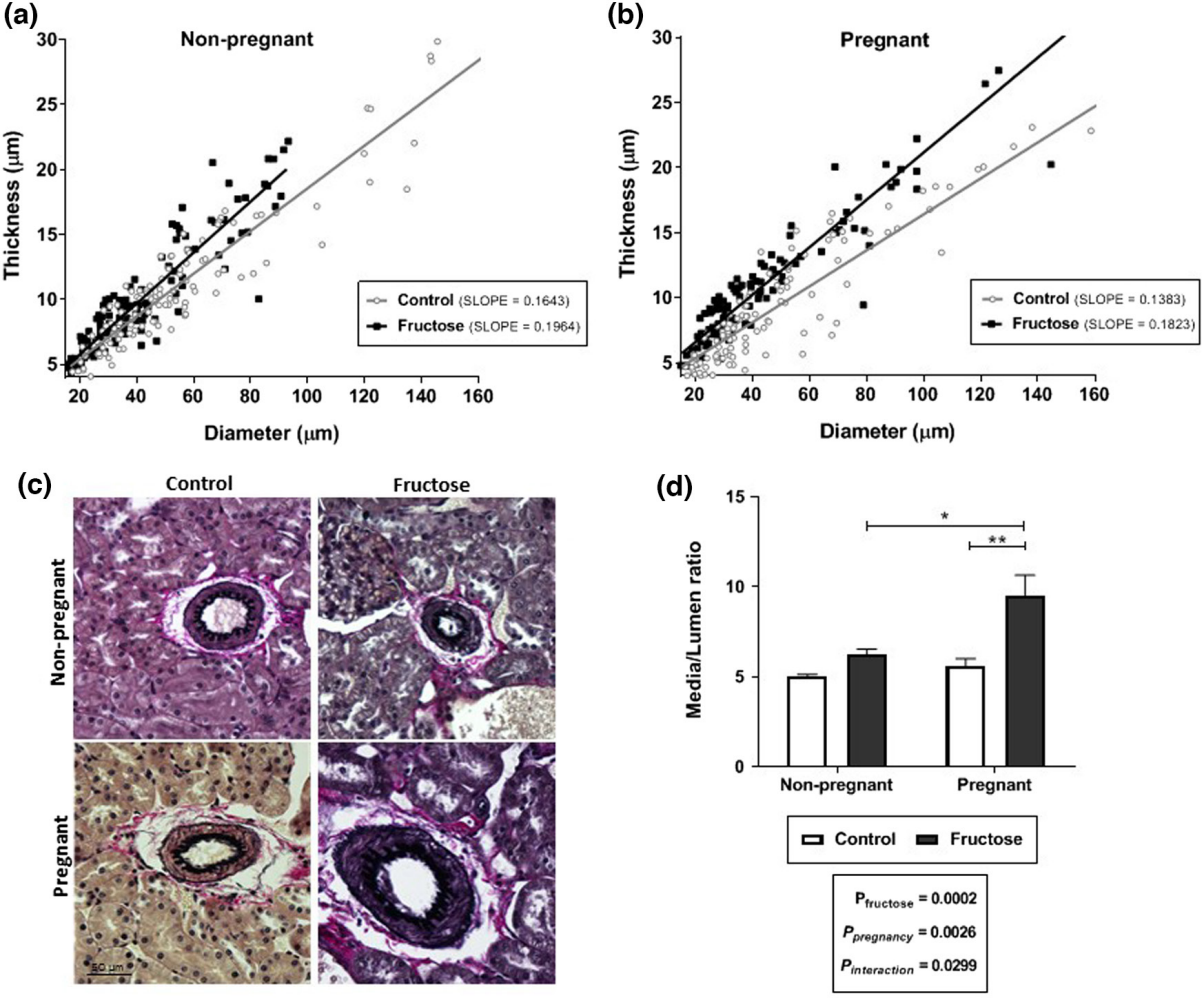
Morphological analysis of interlobular arteries from female rats receiving or not receiving fructose overload. Correlation between thickness and diameter in non‐pregnant rats (a) and pregnant rats (b). Representative photomicrographs of interlobular arteries (original magnification 400×) (c), and media/lumen ratio (d) Summary statistics: Pearson's correlation index: NPC: *r* = 0.9419; NPF: *r* = 0.9185; PC: *r* = 0.8984; PF: *r* = 0.9659. Comparison between fructose and control groups slopes (linear regression; NP: *p* = 0.000631; F = 11.9851; DFn = 1, DFd = 249; NP: *p* < 0.0001; F = 33.1772; DFn = 1, DFd = 233). Media/lumem ratio: two‐way ANOVA & Tukey's post‐hoc test. The assessments were performed one week after weaning on 5–6 animals per group.

The kidney morphological parameters are shown in Figure 2. Pregnancy had a positive effect on the glomerular area (glomerular area in μm^2^: NPC: 7257.9 ± 241 [*n* = 5]; NPF: 6751.3 ± 249 [*n* = 5]; PC: 7427.5 ± 164 [*n* = 5] PF: 7786.9 ± 351 [*n* = 5]; Figure 2b), altering the profile of glomerular area distribution in pregnant rats (Figure 2a). Fructose treatment augmented macrophage infiltration (Figure 2c), alpha‐SM‐actin (Figure 2d), and 8‐OHdG expression (Figure 2f). The eNOS expression was reduced in the fructose‐treated groups (Figure 2e). The number of PCNA positive cells was similar among the groups (Figure 2b). The morphology of the interlobular artery was evaluated in slides stained using the Verhoeff‐Van Gieson method (Figure 3). The correlations between interlobular artery thickness and diameters for pregnant and non‐pregnant groups are presented in Figure 3a,b. The index of correlation was significant for all groups. However, significant changes were observed in the slope of the rats receiving fructose, compared to the controls. Representative illustrations of interlobular arteries are shown in Figure 3c. The vascular cross‐section area (CSA) and the ratio between media/lumen (M/L) were evaluated to obtain more detailed information on the effect of fructose on interlobular arteries, as shown in Figure 3d. Values of CSA in μm^2^ were: NPC: 1205.8 ± 171 [*n* = 5]; NPF: 820.0 ± 69 [*n* = 5]; PC: 1190.8 ± 213 [*n* = 5]; PF: 1120.1 ± 167 [*n* = 5]; there were no significant differences between the groups in respect of CSA. Higher M/L ratio was observed in the fructose‐treated groups in comparison to control group. Higher M/L ratio without a raise in CSA characterizes a eutrophic remodeling (Intengan & Schiffrin, 2000).

## 4 DISCUSSION

In the present study, we observed that fructose overload during pregnancy and lactation in female rats resulted in important changes in renal function, including decreased glomerular filtration rate (GFR) of about 30%; reduced excretion of sodium, potassium, and urea, and increased urinary flow. Fructose also increased blood pressure in pregnant (about 10%) and non‐pregnant (about 15%) rats. These results confirm the association between renal changes and the development of hypertension caused by fructose overload

Hypertension induced by fructose has been related to chronic activation of the sympathetic nervous system (SNS) (De Angelis et al., 2012). This hypothesis is supported by studies that have investigated in rats with high fructose intake the autonomic effects (Bernardes et al., 2018; Tran et al., 2014). Renal sympathetic nerve activation (RSNA) is known to alter renal hemodynamic and tubular sodium and water reabsorption. Additionally, it stimulates renin release and activates the RAS which also favors the elevation of blood pressure (DiBona, 2001; Komnenov et al., 2019). Cabral et al. (2014) observed that in luminal perfusion fluid ANG II increases the effect of fructose on NHE3 activity, suggesting that fructose increases proximal tubule sensitivity to ANG II, augmenting renal sodium reabsorption (Cabral et al., 2014). Increased expression of the NHE3 exchanger and of the Na/K/2Cl cotransporter (NKCC2) was also found by Xu & Yang, 2018 who associated the changes observed with an increase of prorenin receptor expression. Yokota et al., 2018 showed that kidneys of rats fed with fructose presented an increased concentration of Ang II, suggesting that fructose stimulated intrarenal components of the RAS. The reduction in food/sodium intake may also contribute to the activation of RAS (Graudal et al., 2021). Thus, fructose seems to increase renal tubular sodium transport, due to the ANGII and/or RSNA effect, resulting in sodium retention and augmented blood pressure (Cabral et al., 2014; Gonzalez‐Vicente et al., 2018; Komnenov et al., 2019; Soleimani & Alborzi, 2011).

Taking into account the fact that SNS can influence renal function by both direct action, as well as by the activation of the RAS (DiBona, 2000; DiBona, 2001; Sata et al., 2018), it is possible that increased RSNA and augmented RAS activity contributed equally to the changes in GFR observed in the present study. The RSNA, due to its effect in renal vasculature reduces the renal blood flow via stimulation of alpha1‐adrenoreceptors and may reduce GFR (DiBona, 2000). Additionaly, inappropriated activation of angiotensin II may alter afferent and efferent arteriolar resistance decreasing renal blood flow and GFR (Navar, 2014). The role of these mechanisms should be examined in further experiments. Furthermore, the activation of these systems during pregnancy may predispose to hypertension, increasing the risk of adverse maternal and fetal outcomes (Malha et al., 2018; Spradley, 2019).

Regarding the reduced potassium excretion, other mechanisms may be involved in this effect. Fructose intake increased urine volume and reduced potassium excretion in both groups treated with fructose. The increase in urine flow is a change known to increase urinary potassium loss. Thus, at the beginning of treatment, potassium loss must have occurred; however, over time, adjustment mechanisms should have been activated to reduce potassium loss. The reduction of the potassium content in the body seems to stimulate the reabsorption of sodium by the Na^+^/Cl^−^ co‐transporter (NCC) in the distal tubule. As a result, a smaller amount of sodium reaches the collecting duct, reducing sodium reabsorption and potassium secretion in the collecting duct (McDonough & Youn, 2017). It is possible that this mechanism contributed to reduce potassium losses; however, further experiments are needed to confirm this hypothesis.

The fructose intake also reduced renal urea excretion. In the non‐pregnant fructose group (NPF), both blood urea concentration and urinary excretion of urea fell. Access to fructose solution increases water intake, which in turn reduces the release of the anti‐diuretic hormone (ADH) by the pituitary. ADH not only stimulates water reabsorption in the distal nephron but also stimulates urea reabsorption in the medullary collecting duct (Deen et al., 1994); therefore, a fall in plasma ADH increases urea excretion. This may explain the reduction in blood urea concentration observed in the NPF group. On the other hand, hepatic urea synthesis seems to be reduced in the presence of fructose, which could contribute to the reduction of blood urea values in the groups that received fructose (Fauste et al., 2020). However, in the PF group, urea blood concentration was significantly increased in comparison to the NPF group, probably as a consequence of the reduced glomerular filtration rate.

Protein excretion did not change in the present study. Increased urinary protein excretion is a sign of glomerular dysfunction, although tubular changes may also contribute to this finding (Gorriz & Martinez‐Castelao, 2012). A more detailed analysis should be performed to assess fructose‐induced glomerular changes.

Morphological analysis showed an increase in the glomerular area in PC and PF groups. This change may be related to alterations that occurred during pregnancy (increased GFR, cardiac output, and renal blood flow) (Smyth et al., 2013) and that progressively recede after delivery (Kaze et al., 2014).

Morphological changes such as glomerular hypertrophy and inflammatory infiltrate are commonly observed in spontaneously hypertensive rats (SHR) (Kaze et al., 2014); in these animals, there is also vascular remodeling with a decrease in arterial lumen and an increase in vascular resistance (Intengan & Schiffrin, 2000). In the present study, pregnant rats that received fructose (PF) in addition to glomerular enlargement and macrophage infiltration showed an increase in the medium/lumen ratio, suggesting a vascular remodeling process, which may have impacted renal hemodynamics and glomerular filtration rate.

Several mechanisms modify renal hemodynamics such as increased sympathetic activity, activation of the RAS, production of pro‐oxidant and vasoconstrictor substances, among others. Renal sympathetic activation increases renal vascular resistance, and activates RAS, which, through angiotensin II, also increases renal vascular resistance. Furthermore, this peptide, through its binding to the AT1 receptor, promotes the production of reactive oxygen species (ROS), which in turn impairs the production of nitic oxide (NO) and also acts in respect of inflammation and fibrosis (Ratliff et al., 2016; Sachse & Wolf, 2007). The uric acid production due to fructose intake also increased the production of reactive oxygen species (ROS) (Choi et al., 2010). This molecule is considered an antioxidant substance in plasma; however, inside the cell it behaves as a pro‐oxidant (Sautin et al., 2007), increasing the concentration of ROS through the activation of NADPH oxidase. This results in a reduction in the activity of the endothelial NO synthetase enzyme (eNOS) and in the production of nitric oxide (NO) (Ejaz et al., 2007). Another vasoconstrictor substance whose production is increased by fructose overload is endothelin. The increased expression of this peptide appears to be associated with fructose‐induced hyperinsulinemia (Klein & Kiat, 2015). Thus, it is possible that many of these mechanisms are involved in the alterations found in the present work. This hypothesis is corroborated by our observations of increased macrophage infiltration and alpha‐SM‐actin expression (suggesting fibroblast transdifferentiation) as well as the increase in oxidative stress, evidenced by the expression of 8‐OHdG and by the decrease in the expression of the eNOS enzyme.

The results obtained in this study show aspects of renal function modified by fructose overload that lead to renal dysfunction and arterial hypertension. These changes may become permanent and have important repercussions in the future.

## 5 CONCLUSIONS

Our data indicate that the association of high fructose intake with pregnancy aggravated kidney changes resulting in renal dysfunction and hypertension. The changes were observed after weaning, suggesting a risk of long‐term harmful effects.

## AUTHOR CONTRIBUTIONS

L.M.M. and C.F.B contributed equally to the study. C.F.B, L.M.M., D.C.K.L. and G.N.G. conceived and designed the study; C.F.B, D.C.K.L., L.M.M., and R.A. performed the experiments; C.F.B, D.C.K.L. and G.N.G. analyzed the data and interpreted the results of the experiments; D.C.K.L. prepared the figures; C.F.B, and L.M.M. performed the histological analysis. D.C.K.L. and G.N.G. drafted, edited, and revised the manuscript. All authors approved the final version of the manuscript.

## FUNDING INFORMATION

This study was supported by the Fundação de Amparo à Pesquisa de São Paulo – (FAPESP, 2018/03511–0).

## CONFLICT OF INTEREST

The authors declare there are no competing interests.

## ETHICS STATEMENT

The animal study was reviewed and approved by the Ethical Research Committee (CEUA) of the Universidade Federal de Sao Paulo (protocol 7647020614).

## Data Availability

The authors declare that they have full access to all the data in this study, take responsibility for the data integrity and the accuracy of the data analysis. All data are archived in an appropriate public university repository in accordance with publishing policies, and can be accessed through the following link: https://repositorio.unifesp.br .
